# Co-occurrence of viruses and mosquitoes at the vectors’ optimal climate range: An underestimated risk to temperate regions?

**DOI:** 10.1371/journal.pntd.0005604

**Published:** 2017-06-15

**Authors:** Marcus S. C. Blagrove, Cyril Caminade, Elisabeth Waldmann, Elizabeth R. Sutton, Maya Wardeh, Matthew Baylis

**Affiliations:** 1 Department of Epidemiology and Population Health, Institute of Infection and Global Health, University of Liverpool, Liverpool, United Kingdom; 2 National Institute of Health Research Health Protection Research Unit in Emerging and Zoonotic Infections, University of Liverpool, Liverpool, United Kingdom; 3 Department of Medical Informatics, Biometry and Epidemiology, Friedrich-Alexander-Universität Erlangen-Nürnberg, Erlangen, Germany; 4 Department of Zoology, University of Oxford, Oxford, United Kingdom; University of California, Davis, UNITED STATES

## Abstract

Mosquito-borne viruses have been estimated to cause over 100 million cases of human disease annually. Many methodologies have been developed to help identify areas most at risk from transmission of these viruses. However, generally, these methodologies focus predominantly on the effects of climate on either the vectors or the pathogens they spread, and do not consider the dynamic interaction between the optimal conditions for both vector and virus. Here, we use a new approach that considers the complex interplay between the optimal temperature for virus transmission, and the optimal climate for the mosquito vectors. Using published geolocated data we identified temperature and rainfall ranges in which a number of mosquito vectors have been observed to co-occur with West Nile virus, dengue virus or chikungunya virus. We then investigated whether the optimal climate for co-occurrence of vector and virus varies between “warmer” and “cooler” adapted vectors for the same virus. We found that different mosquito vectors co-occur with the same virus at different temperatures, despite significant overlap in vector temperature ranges. Specifically, we found that co-occurrence correlates with the optimal climatic conditions for the respective vector; cooler-adapted mosquitoes tend to co-occur with the same virus in cooler conditions than their warmer-adapted counterparts. We conclude that mosquitoes appear to be most able to transmit virus in the mosquitoes’ optimal climate range, and hypothesise that this may be due to proportionally over-extended vector longevity, and other increased fitness attributes, within this optimal range. These results suggest that the threat posed by vector-competent mosquito species indigenous to temperate regions may have been underestimated, whilst the threat arising from invasive tropical vectors moving to cooler temperate regions may be overestimated.

## Author summary

Mosquito-borne viruses, such as dengue, are believed to cause over 100 million cases of human disease annually. Current mathematical models that aim to predict risk of virus transmission are generally either highly “mosquito-centric” or “virus-centric”. For virus transmission to occur, conditions need to be suitable for both mosquito and virus: hence, we propose a novel approach that considers the interplay between the different optimal conditions for the mosquito and the virus. Our findings indicate that warmer- or colder- adapted mosquitoes are significantly more efficient vectors in warmer or colder climates respectively. Consequently, we propose that there is currently an underestimation of risk to temperate regions from their native and cooler adapted mosquitoes.

## Introduction

Mosquito-borne viruses are an increasing global health problem, predominantly in developing tropical countries, where billions of people are at risk of disease. For example, the World Health Organisation estimates that there are between 50–100 million cases of dengue each year, and the problem is continuing to worsen [1]. Furthermore, over the last few decades, people in the developed world are also becoming increasingly at risk from many arboviruses; for example, West Nile virus has become endemic in the USA [2], and appears to be spreading within Europe [3]. Additionally, recently discovered mutations in the chikungunya virus may have increased its potential for transmission in the more temperate adapted *Ae*. *albopictus*, which may increase transmission risk in temperate regions in the future [4].

The risk of virus transmission by a competent vector is determined by how many feeds it takes on susceptible hosts in its remaining lifespan after the point at which it becomes infectious. The time to becoming infectious (after the vector has taken an infected blood feed) is termed the extrinsic incubation period (EIP). During the EIP of mosquito vectors, the virus spreads from the gut through the body to the salivary glands, and replicates in order to be transmitted; typically, this process takes several days [5]. Both mosquito longevity and virus EIP are highly sensitive to temperature [5, 6, 7]. An increase in temperature generally reduces both the EIP and longevity, but not necessarily by the same amount. If the EIP decreases more than longevity, more time is available for virus transmission and thus transmission risk will theoretically increase; if longevity decreases more than EIP, less time is available for virus transmission and transmission risk will theoretically decrease. In addition to the effect on longevity, temperature is also known to affect other components of vector capacity such as biting rate and larval density [8, 9]. There is not, therefore, a straightforward relationship between temperature and arbovirus transmission risk; rather, it is largely determined by the complex interplay between the competing effects of temperature on the EIP and components of vectorial capacity including longevity.

Most current mathematical models of arbovirus disease risk identify the suitable ranges for viruses and their vectors separately [10, 11, 12], or identify temperature suitability for the vector within a static set of virus requirements [13, 14]. Here, we consider this dynamic interplay between the opposing effects on virus and vector which highlights that the same virus may have different optimal temperatures in different vectors.

An observable effect of this interplay is that in some cases, vector ranges extend beyond the ranges of the viruses they transmit–usually into cooler areas. For example, *Culex pipiens* extends farther north across North America, Europe and Asia than does West Nile virus (WNV); cooler regions such as these, with vector but no virus, are indicative of a limit created by EIP increasing more than longevity. A possible example of the converse–a vector being found in hotter climates than the virus it transmits–may be the biting midge *Culicoides obsoletus sl*, which is found across Europe and north Africa, and which has transmitted bluetongue virus serotype 8 (BTV-8) extensively in northern Europe, but little or not at all in southern Europe [15], where the Afrotropical *Culicoides imicola* is the dominant vector. *C*. *obsoletus* appears to prefer a cooler climate range [16], with higher densities in northern rather than southern Europe, and the higher BTV-8 transmission in northern Europe suggests that its increased longevity at lower (northern) temperatures may outweigh the increased EIP of BTV-8.

**Fig 1:**
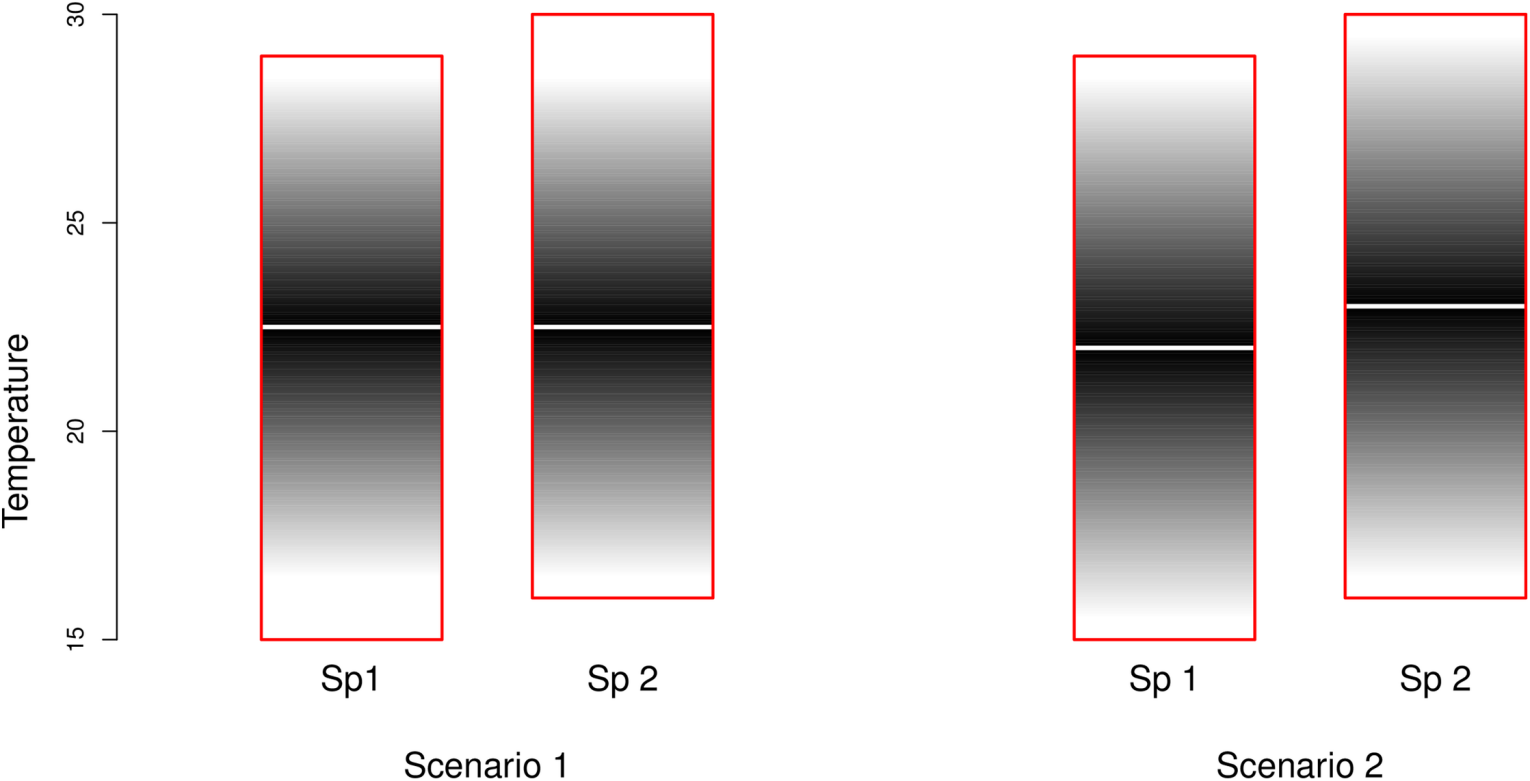
Transmission risk of two example mosquito species. Despite significant overlap in temperature envelopes (red boxes), Species 1 (Sp 1) is more cold-adapted than Species 2 (Sp 2) and its envelope extends further into colder temperatures, and less far into warm temperatures. In scenario 1, the optimum temperature for virus transmission (shown as white line against grey shading) is the same for both Sp 1 and Sp 2, despite the different temperature envelopes of the vectors. Our hypothesis corresponds to scenario 2, where the optimum temperature for virus transmission is lower for cooler-adapted Sp 1, and higher for warmer-adapted Sp 2.

**Fig 2:**
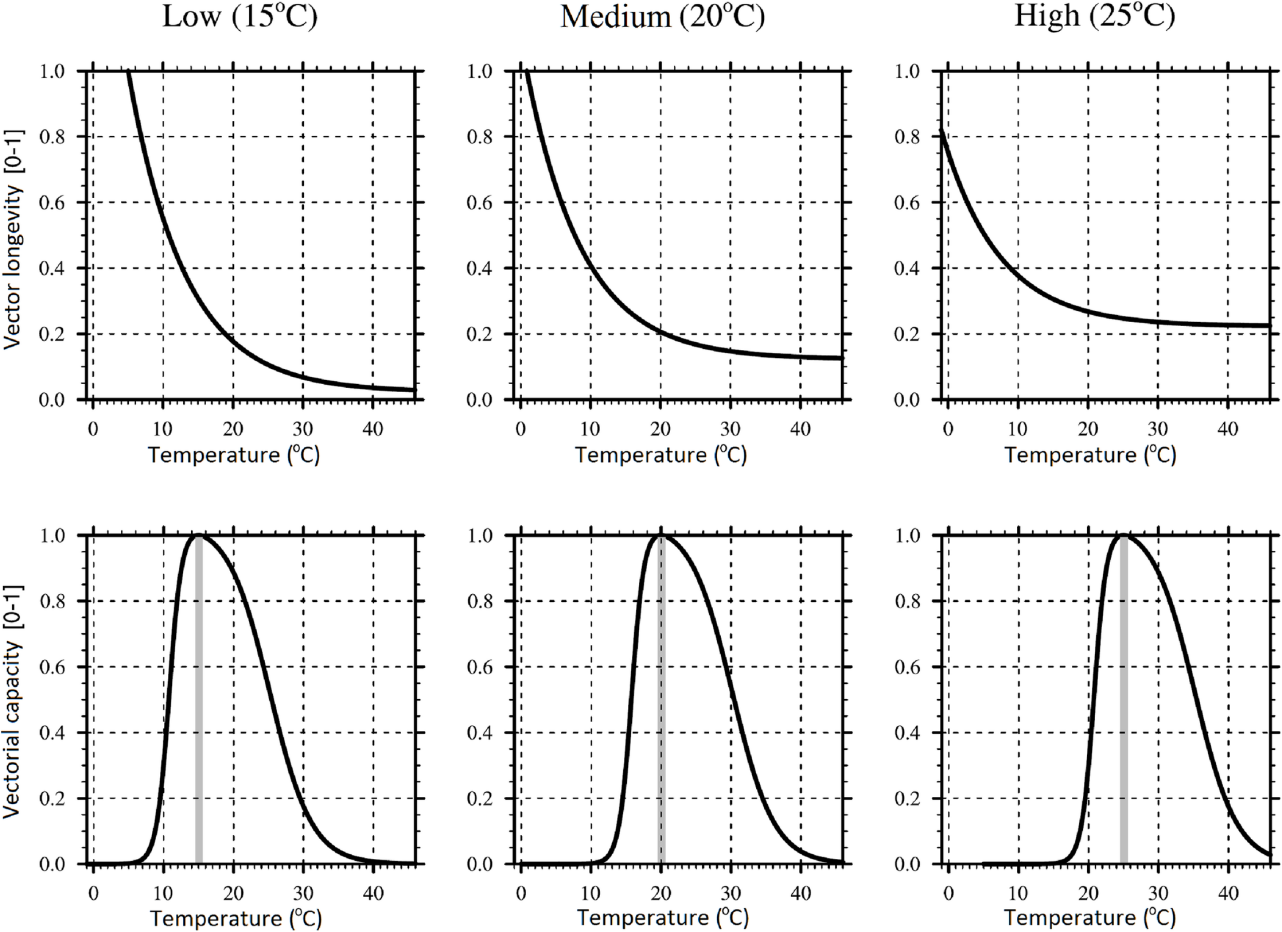
Diagram of the hypothesized effect of temperature on longevity. Longevity of three example mosquito species, each adapted to a different (low, medium and high) mean temperature. As temperature increases, the longevity of all mosquitoes is decreased (top panels). However, here we hypothesize that mosquito species adapted to cooler temperatures will live proportionally longer than will warmer-adapted species in these cooler temperatures and less long in the warmer temperatures (compare top panels). At a given temperature, as longevity increases so does the potential number of infectious bites the vector can make. Consequently, mosquitoes adapted to a particular temperature, will have the highest vectorial capacity at the respective temperature (bottom panels).

These observations led us to hypothesize that the optimal climate conditions for the transmission of an arbovirus by a vector are a compromise between the optimal conditions for the survival of the vector and the effect of the temperature on the EIP of the virus. Here, we apply this hypothesis to the mosquito-borne arboviruses dengue, West Nile and chikungunya viruses. We predict that cooler-adapted mosquitoes have a higher vectorial capacity in a cool climate than would warm-adapted mosquitoes. This higher vectorial capacity towards the optimal temperature conditions of the species in question, we propose, would lead to increased transmission risk in cool areas by cooler-adapted mosquitoes (Fig 1). Underpinning this hypothesis is the assumption that cool-adapted species will have a proportionally greater increase in longevity at colder temperatures than would warmer-adapted species (Fig 2). To test this hypothesis, we used published geolocated data to identify temperature and rainfall ranges in which a number of mosquito vectors are observed to co-occur with a range of arboviruses. We investigated whether different mosquito species have different climatic ranges for co-occurrence with the same virus, and related this to the average temperature ranges of the areas in which the different vector species are found.

## Materials and methods

### Data collection

Species locations, vector-virus relationships, and country and “sub-country” temperature and rainfall data were mined from the ENHanCEd Infectious Diseases (EID2) database (Eid2.liverpool.ac.uk) [17]. We employed the CRUTS3.1 climate dataset, based on monthly means calculated over the period 1950–2000 [18]. “Sub-countries” are defined as first administrative divisions (e.g. states in USA, counties in UK, states in India, prefectures in Japan). This definition is used in the EID2 database and the list of these divisions was taken from: http://www.geonames.org/export/codes.html

EID2 is an open-access and evidence-based web-fronted database that provides a repository of organisms, their interactions (in particular the host-pathogen interactions), and their spatial (at country and sub-country levels), and temporal distributions [17].

EID2 has been built and populated with minimum human intervention, by automatically gathering data from open-access online resources, and then extracting interactions accordingly. This was based on a three-step process:

1. Knowledge curating: The unique identifiers, taxonomic ranks and classifications of 856,031 organisms were obtained from the NCBI Taxonomy database (www.ncbi.nlm.nih.gov/taxonomy/). In addition, to enable the discovery of the geographical distribution of organisms, a comprehensive dictionary of geographical names was built by collecting data from the GeoNames geographical database (www.geonames.org/about.html), and subsequently supplementing it with the list of countries available in the Medical Subject Headings (MeSH) (www.nlm.nih.gov/mesh).
2. Evidence curating: 39,238,061 nucleotide sequences meta-data files, covering the period 1993–2012, were retrieved in XML format from NCBI Nucleotide Sequences database (www.ncbi.nlm.nih.gov/nucleotide). Information regarding the sequenced organisms, and where available their hosts (7.1% of total sequences) and their geographical location (17.53% of total) was extracted. 198,301 of these sequences were from the *Culicidae* family, and in 7,049 files the hosts were from the *Culicidae*. 6,801,436 publications were also retrieved from the PubMed citation index (www.ncbi.nlm.nih.gov/pubmed); of these, 5,433 referred to mosquitoes in the title or abstract.
3. Interaction discovery: Interactions between organisms, and organisms and their locations were extracted from the information gathered using disambiguation and recognition algorithms. Most interactions in EID2 are supported by either: a) nucleotide evidence: host and location information extracted from the sequences’ metadata were mapped to the taxonomy and the geographical names collected in step 1; or b) publication evidence extracted by applying a variety of auto-generated search terms to retrieve publications’ metadata from PubMed and intersecting the results. EID2 uses a threshold of a minimum of five publications for interactions not supported by nucleotide evidence. Additionally, country and sub-country search terms were also applied and the resulting publications were intersected with those of the organisms to provide additional evidence of the geographical distribution of these organisms. EID2 contains 127,576 organism-organism interactions (844 of these are interactions with at least one member of the C*ulicidae*) and 364,142 organism-location interactions (3,712 of which are from the *Culicidae*).

EID2 automatically retrieves data from NCBI PubMed and Nucleotide Sequences Database, and as such relies entirely on the meta-data provided at submission to identify the sub-species. Of the five mosquito and three virus species used in this study, only dengue virus had a practical proportion of the data described to sub-species level (56.1% of data entries); all other species had less than 3% of their data entries described to sub-species level. Consequently, it was decided to use species level, as the sub-species sample size would be too small for meaningful analysis.

### EID2 data validation

Occurrence and absence data from the EID2 database (at the sub-country level) was validated against external observed published datasets. For this validation we used the data for *Ae*. *aegypti*, *Ae*. *albopictus* and DENV as these are the most well studied and have relevant recent publications. For *Ae*. *albopictus* and *Ae*. *aegypti* we compared the EID2 data with point occurrence data published by Kraemer et al. [19]. The dengue virus occurrence data was compared to data published by Bhatt et al. [20].

### Software

Statistical analysis was performed using the R software. Maps were generated using EID2 database grid information (25km x 25km grid with each square associated with a country and where possible a sub-country, or water body) and custom C# code to assign colours to the squares.

### Optimal mosquito season

The temperature experienced by a mosquito vector in a given location (sub-country) varies throughout the year, and it is not straightforward to capture this in a single variable. For our purposes, we first define the four-month period of the year in each sub-country when adult vectors are most likely to be active, based on temperature and rainfall data, and then we extract the mean temperature of those four months only. We call these four months the Optimal Mosquito Season (OMS).

To find a sub-country’s OMS we used both monthly average temperature and rainfall data. We selected the warmest consecutive four-month period (months 1 to 4) which also had at least 24% of the sub-country’s total annual rainfall within a period of four months starting from the preceding month (months -1 to 3). For example, if the warmest four-month period was March to June, this would be the OMS only if the total rainfall in February to May was at least 24% of the total annual rainfall. If not, the next warmest four-month period was checked for appropriate rainfall. The one month rainfall offset is to account for much of the larval development (for which rainfall is most important) occurring before and very little larval development occurring towards the end of the mosquito season. The above parameters were chosen as they appear to result in the best fit of the model with general published observations on mosquito seasons around the world (e.g. www.nhstateparks.com/mosquitos.html, http://www.fitfortravel.nhs.uk/destinations/, www.mosquitoreviews.com); as well as published surveys (for Thailand [21], Ivory Coast [22], Republic of Korea [23] and Brazil [24]). Given the lack of published models or databases for mosquito activity by month, our validation was restricted to manually comparing to published sources.

### Vector temperature ranges

The mean OMS temperature for all sub-countries that a vector species inhabits was determined in order to estimate the optimal temperature for each vector species.

### Vector/Virus co-occurrence temperature ranges

West Nile virus (WNV), chikungunya virus (CHIKV) and dengue virus (DENV) were chosen for analysis to provide a range of globally important viruses transmitted by a wide variety of mosquito vectors. The list of known and potential vectors, as defined by the ENHanCEd Infectious Diseases (EID2) database, for each virus is shown in Table 1. Selection criteria for further analysis were volume of data available for analysis (>10 sub-countries in which it is the sole vector species (whether virus is present or not), and >3 sub-countries in which it is the sole vector and co-occurs with the virus).

**Table 1:** Known and potential vectors of West Nile virus, chikungunya virus and dengue virus listed in the EID2 database.

West Nile virus	Chikungunya virus	Dengue virus
*Aedes albopictus*	***Aedes aegypti***	***Aedes aegypti***
*Aedes cinereus*	*Aedes africanus*	***Aedes albopictus***
***Aedes vexans***	***Aedes albopictus***	*Aedes polynesiensis*
*Anopheles messeae*	*Aedes furcifer*	*Aedes scutellaris*
*Anopheles subpictus*	*Aedes vittatus*	
*Culex annulirostris*		
*Culex bitaeniorhynchus*		
*Culex interrogator*		
*Culex nigripalpus*		
***Culex pipiens***		
*Culex pseudovishnui*		
***Culex quinquefasciatus***		
*Culex restuans*		
*Culex salinarius*		
*Culex tarsalis*		
*Culex univittatus*		
*Culex vishnui*		
*Culiseta melanura*		
*Dermacentor marginatus*		
*Hyalomma marginatum*		
*Ochlerotatus canadensis*		
*Ochlerotatus cantator*		
*Ochlerotatus sollicitans*		
*Ochlerotatus sticticus*		
*Ochlerotatus triseriatus*		
*Psorophora ferox*		

The requirement for the mosquito to be the sole vector species in analysed sub-countries was because it was assumed that for these sub-countries, the vector in question is likely to be responsible for most of the virus transmission, and thus analysis could be performed with fewer concerns over contributions to transmission made by other vector species. There were many sub-countries where more than one vector species for the respective virus is recorded in the EID2 database; these records were ignored in our analysis, as it was not possible to ascertain which of the vectors are transmitting the virus in those sub-countries (94/326 for CHIKV; 89/272 for DENV; 179/387 for WNV, numbers include all sub-countries for all vectors, not just vectors used in the analysis here). Whilst we focused on a small number of possible vectors (shown in bold), we defined “sole vector” as being the only recorded vector from the full list, not just from the analysed subset.

For each virus, we then extracted the mean temperature of the OMS for every sub-country for which the EID2 database has recorded the presence of a single vector species for that virus. For each vector species for each virus, OMS temperatures were compared between sub-countries with vector only and sub-countries with vector/virus co-occurrence.

The OMS temperature ranges were then compared statistically to examine whether two or more vectors co-occur with the same virus at significantly different OMS ranges, whilst accounting for the different temperature ranges at which each vector occurs (i.e. does a cooler-adapted vector co-occur with virus more frequently at the cool end of its range vs. a warmer-adapted vector?). This was achieved by standardizing the temperature data for each vector distribution, before conducting the test as described below.

Assume mosquito *j* lives in *n*_*j*_ different sub-countries, then the standardised temperatures:

t*_ij_, *i* ∈ (1…, *j*) for this sub-country are:

$$t_{ij}^{*}=\frac{t_{ij}-{\bar{t}}_{j}}{\mathrm{sd}(t_{j})},$$

where ${\bar{t}}_{j}$ is the mean and sd(*t*_*j*_) the standard deviation of the raw set of temperatures *t*_*ij*_.

Following standardisation, each vector’s sub-country-level temperature data have means of zero and standard deviations of 1. Two-tailed Wilcoxon rank-sum tests were then performed on the subset of standardised data for the sub-countries containing both virus and vector.

### Kernel density estimation

Kernel density estimation was used to estimate the empirical density of mosquito/virus co-occurrence without assuming a known underlying distribution. The underlying formula to estimate the distribution *f* of a variable *X*, of which we have n observations *x*_*1*_,…, *x*_*n*_ is (Eq 1):

$$\hat{f}(x)=\frac{1}{hn}\sum_{i=1}^{}nK(\frac{x-x_{i}}{h})$$ Eq 1

With *K* being an appropriate kernel function and *h* being the bandwith for the evaluation of *K*. In this case a Gaussian kernel was used, i.e. the function *K*(*u*) is (Eq 2):

$$K(u)=\frac{1}{\sqrt{2\pi }}\exp(-\frac{u^{2}}{u})$$ Eq 2

The bandwidth *h* was calculated adaptively. For the subsets of the data the estimated density was multiplied by the proportion of data in each subset.

## Results

### Validation of EID2 data

See supplementary materials (S1, S2 and S3 Figs and S1 Table) for details about EID2 data validation; briefly, 89.8%, 91.8% and 70.6% of observational points (as published in [19, 20]) were inside EID2 polygons for *Ae*. *aegypti*, *Ae*. *albopictus* and DENV respectively.

### Optimal mosquito season

**Fig 3:**
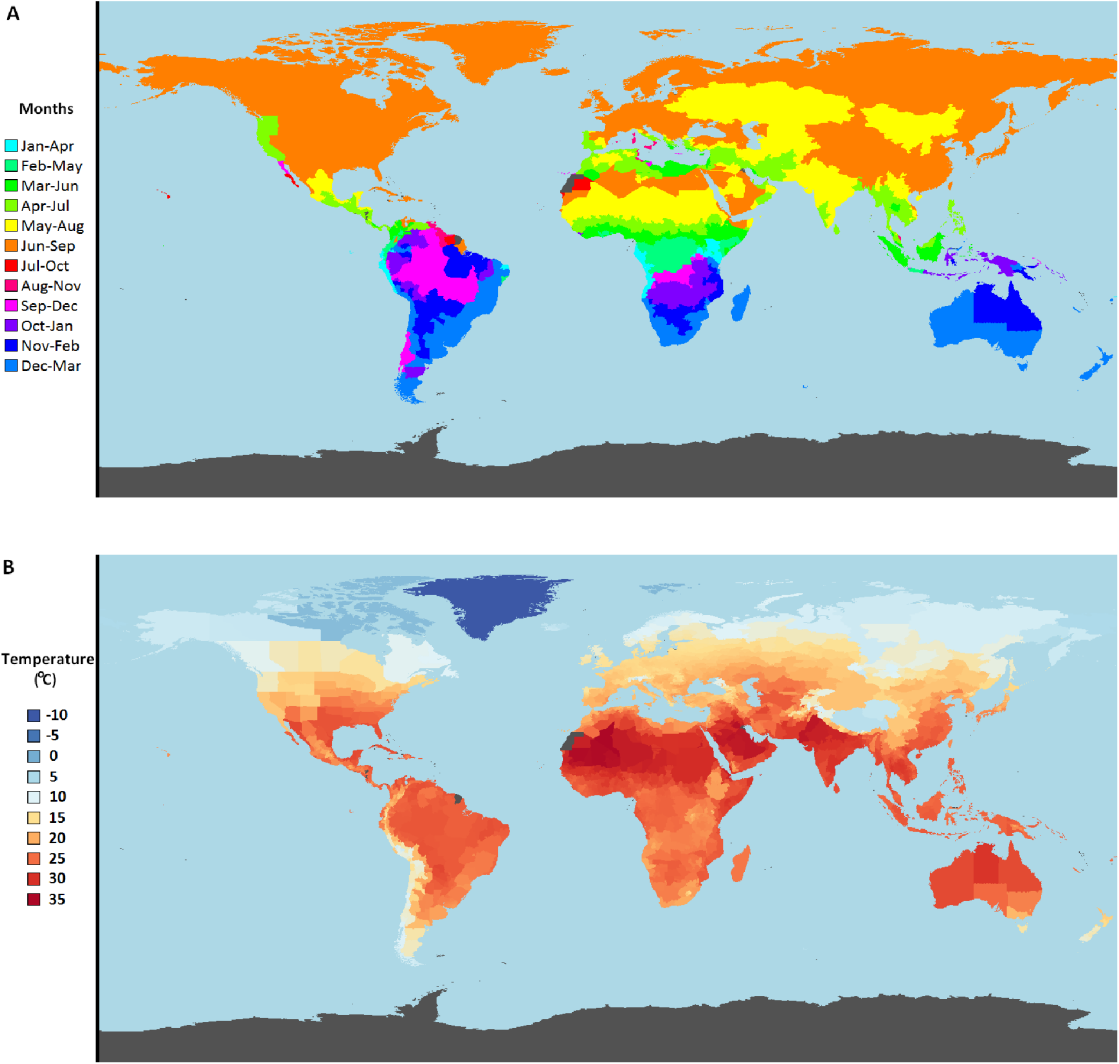
Optimal mosquito season for sub-countries. Maps showing A: the period of the OMS as defined by the model parameters described in the methods; B the mean temperature of this season. Areas coloured in grey have insufficient data for this analysis.

The periods of the OMS as defined by our parameters in different sub-countries are shown in Fig 3A. The mean temperature of the four months was taken and defined as that sub-country’s OMS temperature; the values of this temperature in different sub-countries are shown in Fig 3B. A detailed list of all sub-countries with their OMS months and temperatures can be found in the supporting information (S2 Appendix).

### Vector/Virus co-occurrence temperature range of individual vector species

**Fig 4:**
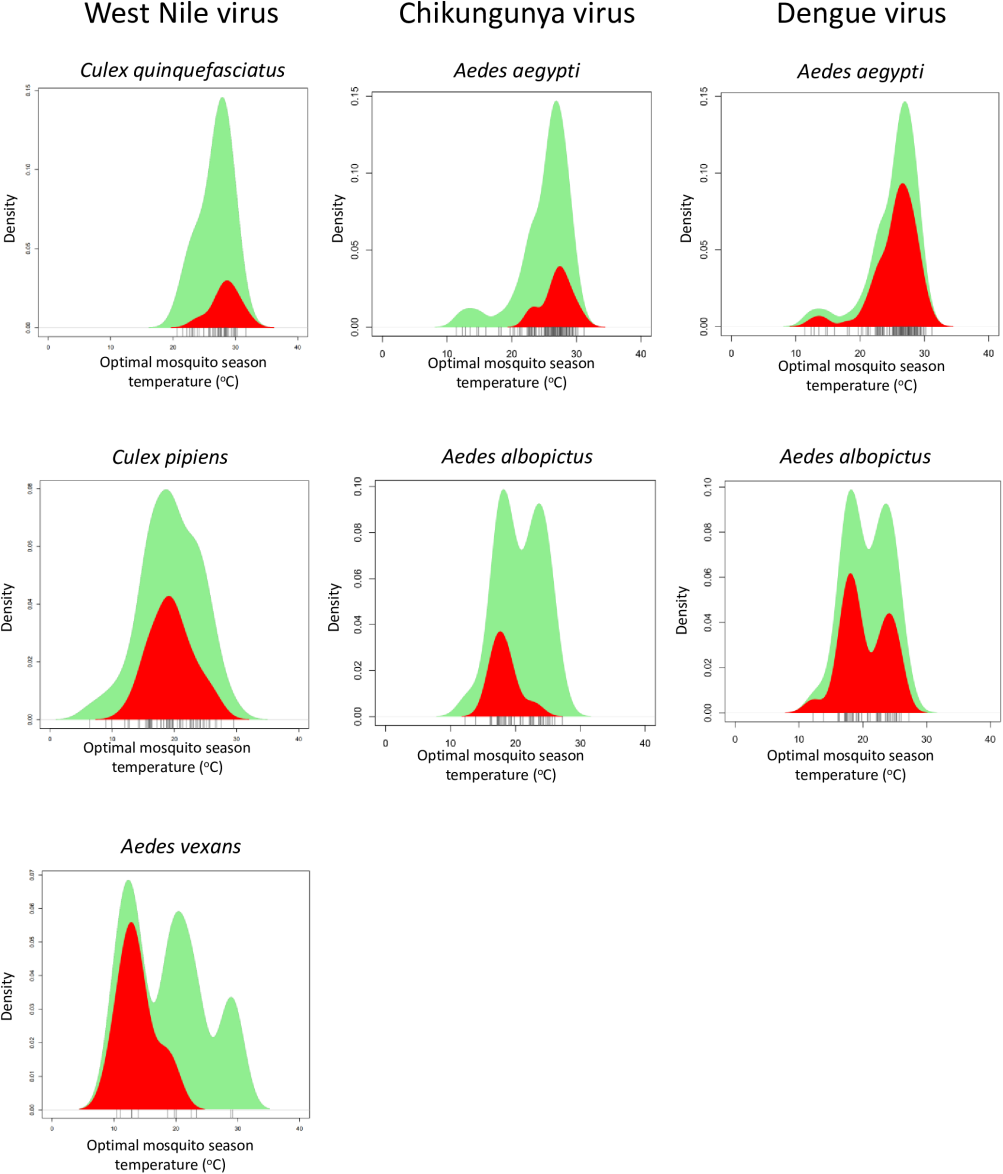
Temperature ranges of vectors and vector/virus co-occurrence. Kernel density estimations for the density of the different mosquitoes across their temperature ranges. The green area represents all single vector sub-countries, whilst the red area represents the density of sub-countries in which the relevant virus was also found. For single vector sub countries with or without virus/ single vector sub countries with virus only: chikungunya virus (*Ae*. *aegypti* n = 112/24, *Ae*. *albopictus* n = 48/9), dengue virus (*Ae*. aegypti n = 117/73, *Ae*. *albopictus* n = 48/24); West Nile virus (*Ae*. *vexans* n = 12/5, *C*. *pipiens* n = 63/25, *C*. *quinquefasciatus* n = 46/9). Grey ticks beneath the graph represent the actual OMS temperature of each of the sub-countries (with or without virus) described by the graph. Note: values of *n* for the same vector species but different viruses vary as a result of different lists of vector species resulting in different numbers of single-vector sub-countries.

For CHIKV, the standardised temperature ranges at which *Ae*. *aegypti* and *Ae*. *albopictus* co-occur with virus were found to be highly significantly different (two-tailed Wilcoxon rank-sum test, p<0.0001), the peak for *Ae*. *albopictus* being at ~18°C, whilst the *Ae*. *aegypti* peak is at ~28°C (Fig 4). For DENV, both *Ae*. *aegypti* and *Ae*. *albopictus* have a peak for co-occurrence with virus at around 24–26°C; while *Ae*. *albopictus* has an additional, and larger, peak at about 18°C. However, overall there was no significant difference (two-tailed Wilcoxon rank-sum test, p = 0.3442). For WNV, three vectors were analysed, therefore two-tailed pairwise Wilcoxon rank-sum tests with Holm-adjusted p-values were performed. A significant difference was found between the standardized temperature ranges of *Ae*. *vexans* and *Cx*. *quinquefasciatus* (p = 0.0068). No significant difference was found between pairwise comparisons of either *Cx*. *pipiens* and *Cx*. *quinquefasciatus or Cx*. *pipiens* and *Ae*. *vexans* (both p = 0.0806) due to overlap between their ranges. However, taken together, the data appear to show a descending order of temperature range of 1) *Cx*. *quinquefasciatus*, 2) *Cx*. *pipiens*, 3) *Ae*. *vexans*.

### Vector temperature range

In order to estimate the optimal temperature for each vector species, the mean OMS temperature for all sub-countries that a vector species inhabits was determined. These temperatures are shown in Table 2. The mean OMS temperature of single-vector sub-countries for each of these vectors are shown to identify any bias in the use of single-vector sub-countries. For four of the five vectors there was no significant difference between the two temperatures (Wilcoxon rank-sum tests); *Ae*. *albopictus* however, shows a significant decrease in temperature for mean single-vector sub-country OMS (P<0.001).

**Table 2:** Vector mean OMS temperatures.

Vector species	Mean OMS temperature	Mean single-vector OMS temperature	Statistical difference (Wilcoxon rank-sum)
*Ae*. *aegypti*	25.6°C (SD = 3.67°C)	25.0°C (SD = 4.24°C)	P = 0.171
*Ae*. *albopictus*	24.4°C (SD = 4.00°C)	20.7°C (SD = 3.51°C)	P<0.001
*Ae*. *vexans*	20.0°C (SD = 4.68°C)	18.6°C (SD = 6.52°C)	P = 0.447
*Cx*. *pipiens*	20.0°C (SD = 4.25°C)	19.3°C (SD = 4.70°C)	P = 0.331
*Cx*. *quinquefasciatus*	26.1°C (SD = 3.30°C)	26.7°C (SD = 2.77°C)	P = 0.238

The mean OMS temperatures were compared for species that are vectors of the same virus; allowing identification of pairs of vectors that have both a difference in their virus co-occurrence temperature range and a difference in their optimal vector temperature range. For the CHIKV and DENV vectors *Ae*. *aegypti* and *Ae*. *albopictus*, there was found to be a significant difference in the mean OMS temperatures (two-tailed Wilcoxon rank-sum test, p = 0.0031). Again, two-tailed pairwise Wilcoxon rank-sum tests with Holm-adjusted p-values were performed for the three vectors of WNV. No significant difference between the mean temperatures of *Cx*. *pipiens* and *Ae*. *vexans* was found (p = 0.95); however, highly significant differences were found between the mean OMS temperatures for *Cx*. *pipiens* and *Cx*. *quinquefasciatus*, and *Ae*. *vexans* and *Cx*. *quinquefasciatus* (both p<0.0001).

## Discussion

We have shown that different mosquito vectors appear to co-occur with the same virus at different temperatures, despite extensive overlap in the temperature ranges occupied by the vectors themselves. For example, our data show that *Ae*. *albopictus* is the primary vector for CHIKV in Regione Lombardia, Italy, which has an average OMS temperature of 17.3°C; this is outside of the OMS temperature range in which we observe *Ae*. *aegypti* as the sole CHIKV transmitter, but well within the observed OMS temperature range of the vector. Conversely, the State of Andhra Pradesh, India, whose average OMS temperature is 29.8°C, is shown here to have CHIKV primarily transmitted by *Ae*. *aegypti*; again, this OMS temperature is above that in which we observe *Ae*. *albopictus* as the sole vector, but within the vector’s own habitable range. Furthermore, we show that this pattern is broadly in line with that of the mean temperatures of the ranges the vectors inhabit–in other words, species that are found, on average, in cool regions tend to co-occur with virus at the cool end of their temperature range.

Taken together, the data presented here suggest that mosquitoes are more competent vectors in climates that are more similar to those which they are adapted to. That is, the same virus can have different optimal temperatures/climates depending on the vector in question. We hypothesise that this effect is primarily the result of a proportionally greater increase in vector longevity versus EIP duration in the vectors’ preferred climate, as well as the effect of temperature on other components of vector capacity such as vector density. Thus, whilst great care must be taken when extrapolating from these results, we further suggest that the current perceived threat of invasive tropical vectors to temperate regions might be an over-estimation. Conversely however, we suggest that lab-competent mosquitoes native to temperate regions [25, 26] potentially pose a much greater risk to temperate regions than is currently perceived, primarily due to their adaptation to their native environment. We believe that this is the first study to consider the interplay between the optimal climates for vector and virus using observational data; our results show a distinct difference between these optimal transmission climates.

In this study we have used the EID2 database to look at sub-countries with records of only a single vector for a given virus. It was assumed that for these sub-countries, the vector in question is likely to be responsible for all or most of the virus transmission, and thus analysis can be performed with fewer concerns over contributions to transmission made by other vector species. All data procured from EID2 were manually spot-checked for accuracy by checking the evidence paper and/or sequence file. Unexpected data were verified, corrected or deleted e.g. removal of false positive species location data such as WNV presence in England and Wales (in this particular example, EID2 reported the presence of WNV in England and Wales as a result of multiple papers reporting the *absence* or *potential* of WNV in these countries). With the exception of a misclassification error (‘*Culex pipiens fatigans*’ appearing when searching for ‘*Culex pipiens*’ and not appearing when searching for ‘*Culex quinquefasciatus*’), only eight out of the 1000 spot-checked sub-country calls were found to be inaccurate. In addition, the location data for *Ae*. *aegypti*, *Ae*. *albopictus* and DENV were validated to recently published data sets (see supplementary materials).

Despite the statistical significance of our results, we acknowledge certain limitations to the data and analyses used here that restrict our ability to draw firm conclusions. In this section we discuss the following limitations to the data and methods:

1. The use of single vector sub-countries only in our analysis.
2. The ‘patchiness’ of data in EID2.
3. The ecological heterogeneity of some sub-countries.
4. The use of OMS to describe climate of sub-countries.

(i) The mean OMS temperature of all sub-countries containing a vector was compared to the mean OMS for single-vector sub countries in order to identify any bias introduced during this ‘sub-sampling’ of the data. For four of the five vectors, there was no significant difference, with only *Ae*. *albopictus* showing a significant change. This is likely the result of the exclusion of sub-countries on the warmer end of the *Ae*. *albopictus* range in which the vector more frequently co-habits with *Ae*. *aegypti* (the larger numbers of single-vector sub-countries containing only *Ae*. *aegypti* appear to have minimised this effect on *Ae*. *aegypti* OMS temperatures). We acknowledge that this represents a possible source of bias in the *Ae*. *albopictus* analyses, which may have biased the results showing *Ae*. *albopictus* to co-occur more towards the cool end of its range. However, we do not think that this is a major concern. Firstly, there is still very significant overlap between the vector temperature ranges of *Ae*. *albopictus* and *Ae*. *aegypti* using single-vector sub-countries, and with the chikungunya data, the majority of co-occurrence of vector and virus is below the temperature where co-occurrence begins for *Ae*. *aegypti*: even if large amounts of data from the overlapping range of *Ae*. *albopictus* has been lost, this peak would still show a marked difference. Secondly, the pattern of vectors found in cool regions transmitting at cooler temperatures is still seen in the three-way comparison for West Nile virus, despite none of these vectors showing skewing of their data.

(ii) A shortfall of the EID2 database is the ‘patchy’ data; some countries and sub-countries are under-represented in terms of sequence uploads to Genbank. Indeed, there is a correlation between wealth of a region (as measured by the gross domestic product or GDP) and the number of sequences it generates and uploads [27]. This, combined with the correlation of cooler climate and wealth, may result in a greater number of cooler sub-countries being analysed. However, this effect is expected to be consistent across all species of vector and virus from within the same sub-country, which would minimise any potential bias. In sub-countries where there are no mosquito species data, these sub-countries are not included in the analysis, minimising the effect of patchy data on the analysis beyond a reduction in statistical power. Furthermore, a large proportion of the sub-countries that do have mosquito species data do so because of NCBI papers and/or sequences pertaining to a general mosquito survey, decreasing any potential problems due to incomplete reporting of the species present in a given sub-country. Consequently, the set of sub-countries where there is assumed to be only one vector is likely to be adequately reliable for the scope of this study. As a result of using only single-vector sub-countries to define virus/vector co-occurrence temperature ranges, a proportion of the data was unusable. A related shortfall is that the majority of data in Pubmed and Genbank does not contain subspecies information; consequently, our analysis was limited to the species level and may have missed important distinctions between different subspecies. This is one possible explanation for the two peaks evident in the temperature range of *Ae. albopictus* (Fig 4), although there may be others. Furthermore, within species there can exist a population structure which correlates with environmental conditions, such as climate. This population structure can result in local adaptations (to both vector and virus) which may make different populations from the same species more suited for transmission in different climates. This cannot be accounted for using the methodology in this study as we have no information on the population structure and associated differences in competence. Instead we were restricted to analyse species as single units with no regard for population structure. Whilst this may have an effect on our results, we theorise that this type of population structure would serve to increase local vector and virus fitness in their respective local climates, consequently, it would likely lead to a non-directional increase in competence throughout all climate ranges and thus would not skew our findings.

(iii) Due to the lack of a finer geo-location scale (as a result of meta-data limitations), sub-country level data were used in our analysis. Whilst this is the finest-scale that could be used for this analysis, a small number of these sub-countries are relatively large (such as Texas, Alaska, Queensland, Western Australia etc.), and there is substantial ecological variation within some of these territories. However, this applies to only a relatively small number of sub-countries and we believe it is unlikely to significantly affect the conclusions. The vast majority of sub-countries are considerably smaller and less ecologically heterogeneous than those mentioned above.

(iv) As mentioned, many current models rely mainly or solely on temperature data for their predictions, and generally consider viruses and their vectors separately rather than considering the interplay between them. Our methodology addresses these issues by defining an optimal mosquito season (OMS) taking into account both temperature and rainfall. In addition, unlike most previous models, our methodology is not limited by laboratory-confined experimental data. All inferences about the climate ranges of vectors and viruses come from published geolocated field data, removing the need for experimental data, which may lack ecological validity, and allowing us to more accurately predict real-world climate ranges of mosquito species. One significant limitation of the OMS method however, is that it produces a single mean temperature for the four-month season, and loses for example any information about variation of temperature and rainfall within the season, which may be important for transmission.

Using the described methodology, our model predicts that for the WNV vectors *Ae*. *vexans* and *Cx*. *quinquefasciatus*, *Ae*. *vexans* has both a significantly cooler ideal temperature range than *Cx*. *quinquefasciatus* and also a significantly cooler range in which it transmits (co-occurs with) the virus. We propose that the cool climate adaptation of *Ae*. *vexans* increases its vectorial capacity at cooler temperatures–possibly via a disproportional increase in longevity compared to EIP–over that of *Cx*. *quinquefasciatus* in such regions. This is in line with previous research showing that the EIP of WNV is very strongly affected by temperature, with the EIP of WNV in *Cx*. *tarsalis* being approximately seven times longer at 14°C than at 26°C [28]. Similar findings are also seen for CHIKV; the two major vectors (*Ae*. *aegypti* and *Ae*. *albopictus*) have highly significantly different temperature ranges, and, the cooler-adapted *Ae*. *albopictus* has a significantly cooler virus transmission temperature range than the warmer-adapted *Ae*. *aegypti*.

However, the same is not seen for DENV, where the transmission temperature ranges of the two vectors (again *Ae*. *aegypti* and *Ae*. *albopictus*) are not significantly different. A possible biological explanation for the lack of an effect for DENV could be the effect of temperature on the EIP of the virus. A recent meta-analysis of the effect of temperature on the EIP of DENV [29] shows a very strong reduction of the EIP with increasing temperature at high temperatures (25–30°C), but little effect of temperature on the EIP at lower temperatures (<20°C). This limited effect on EIP at lower temperatures means that even warm-adapted species, such as *Ae*. *aegypti*, may still be able to transmit the virus at lower temperatures despite the temperature being sub-optimal for the vector; similarly, at high temperatures, the large reduction in EIP means that even cool-adapted vectors such as *Ae*. *albopictus* survive long enough to transmit. Taken together, the EIP-temperature profile of DENV is in line with the wider competent temperature ranges for vectors, particularly outside of their optimal temperatures.

A recent meta-analysis of vector longevity at different temperatures [30] predicts a higher longevity of *Ae*. *aegypti* at high temperatures (~>35°C) than *Ae*. *albopictus*, whilst *Ae*. *albopictus* has a higher longevity at lower temperatures. Given this, along with the CHIKV data and modelling presented here, the evidence is in line with our hypothesis that at their respective optimal temperatures, *Ae*. *albopictus* and *Ae*. *aegypti* live significantly longer and have higher capacity to transmit the virus.

A third temperature-sensitive factor, important in determining the vectorial capacity of a species, is the feeding interval. However, while investigation of the effect on feeding interval is beyond the scope of this study, there is evidence that increasing temperature increases blood-feeding frequency in a range of mosquitoes [31]. This factor could decrease the risk of transmission in cool areas, however, more research would be needed, especially for native temperate mosquito species, as to whether this holds true for cool-adapted species.

Taken together, these data, and the wider literature, are in line with the proposed hypothesis that the optimal climate conditions for the transmission of an arbovirus are due in part to a compromise between optimal conditions for the growth, longevity and competence of the vector and the effect of the temperature on the EIP of the virus. The recent example of BTV-8 transmission by the cool-adapted midge *Culicoides obsoletus sl*, in northern Europe, but not southern Europe, despite being present in both, is consistent with this hypothesis.

To our knowledge, this is the first study using observational data that demonstrates different mosquito vectors have different competent climate ranges for the same virus. For CHIKV and WNV, their respective vectors appear to have a higher competence in temperature ranges to which they are more adapted, suggesting that the threat from arbovirus transmission is greater from vectors native to the respective region (or invasive species from a region with a similar climate). This has important implications in the estimation of risk from vector/virus combinations, especially in more temperate regions which may be at greater risk from competent native temperate vectors than is currently believed.

## Supporting information

S1 Appendix List of all single-vector sub-countries for each virus and vector combination. (XLSX)

S2 Appendix List of all sub-countries with their OMS months and OMS temperatures. (XLSX)

S1 Fig Presence and absence of *Ae*. *aegypti* (all hits from EID2, not just single-vector). The orange regions depict regions where presence was confirmed by the EID2 database. The black points are presence data point based on the work of Kraemer et al. [19].
(TIF)

S2 Fig Presence and absence of *Ae*. *albopictus* (all hits from EID2, not just single-vector). The orange regions depict regions where presence was confirmed by the EID2 database. The black points are presence data point based on the work of Kraemer et al. [19].
(TIF)

S3 Fig Presence and absence of dengue virus. The orange regions depict regions where presence was confirmed by the EID2 database. The black points are presence data point based on the work of Bhatt et al. [20].
(TIF)

S1 Table Number of observational points (from [19, 20]) inside and outside the EID2 database polygons (DOCX)

S1 Materials (DOCX)
