# Supplementary figures and images for: Co-occurrence of viruses and mosquitoes at the vectors’ optimal climate range: An underestimated risk to temperate regions?
Source: PLoS Negl Trop Dis. 2017 Jun 15;11(6):e0005604. doi: 10.1371/journal.pntd.0005604 (PMC5487074; doi:10.1371/journal.pntd.0005604)

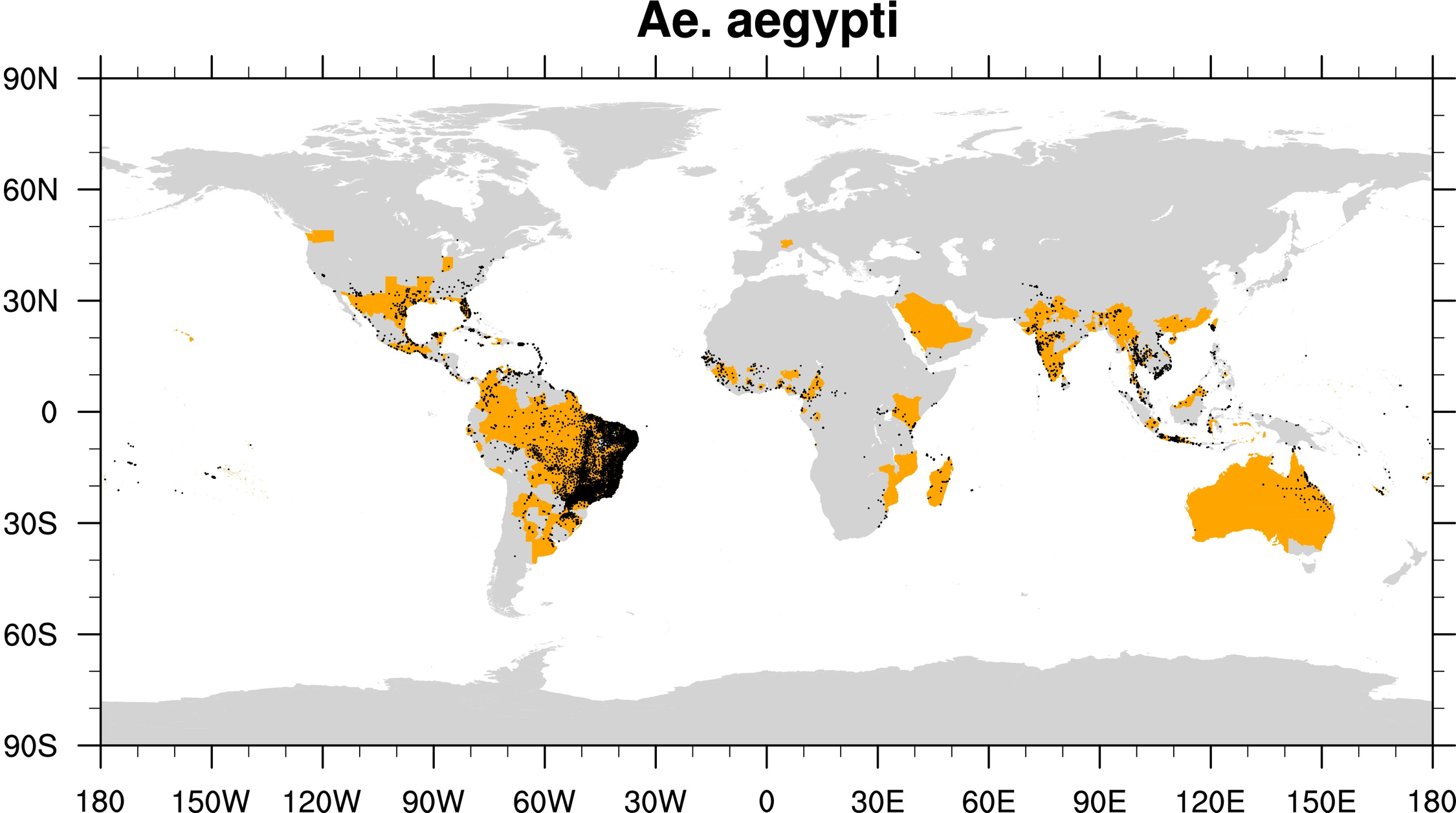

Supplement: S1 Fig — The orange regions depict regions where presence was confirmed by the EID2 database. The black points are presence data point based on the work of Kraemer et al. [19]. (TIF) [file pntd.0005604.s003.tif]

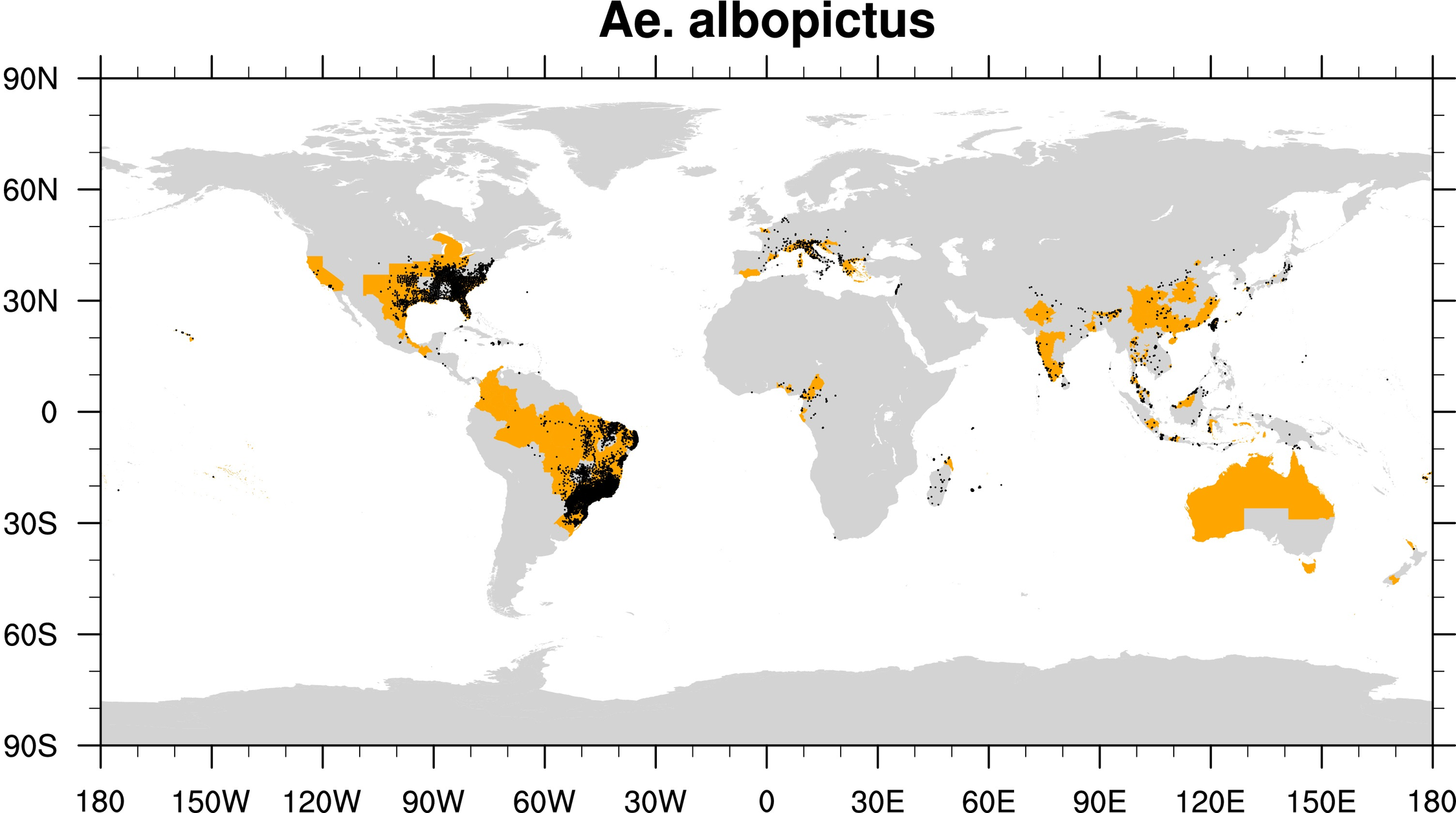

Supplement: S2 Fig — The orange regions depict regions where presence was confirmed by the EID2 database. The black points are presence data point based on the work of Kraemer et al. [19]. (TIF) [file pntd.0005604.s004.tif]

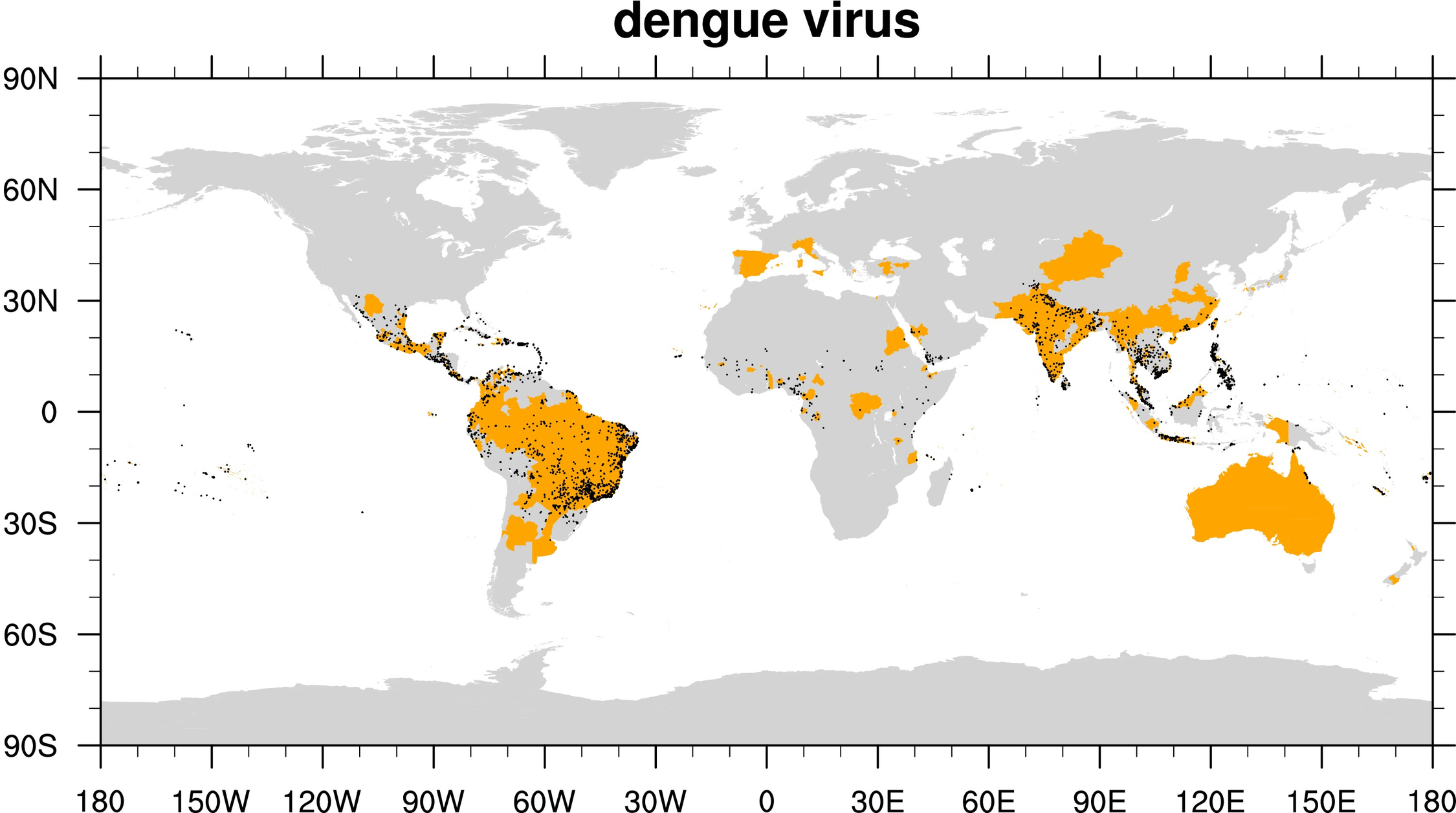

Supplement: S3 Fig — The orange regions depict regions where presence was confirmed by the EID2 database. The black points are presence data point based on the work of Bhatt et al. [20]. (TIF) [file pntd.0005604.s005.tif]
