# Supplementary material for: Co-occurrence of viruses and mosquitoes at the vectors’ optimal climate range: An underestimated risk to temperate regions?
Source: PLoS Negl Trop Dis. 2017 Jun 15;11(6):e0005604. doi: 10.1371/journal.pntd.0005604 (PMC5487074; doi:10.1371/journal.pntd.0005604)
Supplement: S1 Table — (DOCX) [file pntd.0005604.s006.docx]

|  | Number of points inside (n , %) | Number of points outside (n, %) | Total number of points |
| --- | --- | --- | --- |
| *Ae. aegypti* (28) | 17903 (89.8) | 2026 (10.2) | 19929 |
| *Ae. albopictus* (28) | 20331 (91.8) | 1806 (8.2) | 22137 |
| Dengue virus (29) | 5871 (70.6) | 2438 (29.4) | 8309 |
