# Supplementary material for: Co-occurrence of viruses and mosquitoes at the vectors’ optimal climate range: An underestimated risk to temperate regions?
Source: PLoS Negl Trop Dis. 2017 Jun 15;11(6):e0005604. doi: 10.1371/journal.pntd.0005604 (PMC5487074; doi:10.1371/journal.pntd.0005604)
Supplement: S1 Materials — (DOCX) [file pntd.0005604.s007.docx]

**Supplementary materials**

EID2 data validation

The EID2 database properly captures the distribution of *Ae. aegypti* globally (Fig S1). About 90% of the occurrence points (based on observation gathered by Kraemer [19]) are inside the polygon outputs from the EID2 database (table S1). The EID2 data over predicts the presence of *Ae. aegypti* over the Washington state of the USA, over the Rhône-Alpes region in France, over Maluku in Indonesia. The distribution of *Ae. albopictus* is also mainly captured by the EID2 database (Fig S2). 92% of the occurrence points are inside the polygon outputs (table S1). The EID2 data predicts *Ae. albopictus* to be present in Australia, however, this species has not yet been observed in the country. It is likely that EID2 predictions are as a result of large amounts of data stating that there is no *Ae. albopictus* present (including control sequencing data geolocated to these areas). Note that both datasets miss the spread of *Ae. albopictus* over larger regions in Europe (see <http://ecdc.europa.eu/en/healthtopics/vectors/vector-maps/Pages/VBORNET_maps.aspx> for the most up to date surveillance dataset for that particular species).

The distribution of dengue cases is mainly tropical (Fig S3). The EID2 database captures 70% of the occurrence data published by Bhatt [20]. The EID2 over predicts dengue occurrence over China and Australia. Note that both datasets miss recent autochthonous cases of dengue over southern Europe (Croatia, France). The EID2 captures recent autochthonous dengue cases reported in Spain in 2015 (not captured by Bhat [20]), but dengue cases shown over north-western Italy were due to infected travellers returning from endemic countries.
